# Two concomitant thrombotic microangiopathies in the background of systemic lupus erythematosus: A case report

**DOI:** 10.1016/j.amsu.2022.104541

**Published:** 2022-09-01

**Authors:** Abdullah AlGhobaishi, Ahmed Hafez Mousa, Reham Salama Alshaltoni, Amani Sail Mohsen, Abdullah Baothman, Hanan Adem, Yasir Eisa, Abeer Amin, Burhan Edrees

**Affiliations:** a Department of Pediatrics, King Fahad Armed Forces Hospital, Jeddah, Saudi Arabia; b College of Medicine and Surgery, Batterjee Medical College, Jeddah, Saudi Arabia; c Department of Pediatrics, Saudi German Hospital, Jeddah, Saudi Arabia; d King Saud Bin Abdulaziz University for Health Science, Jeddah, Saudi Arabia; e Department of Pediatrics, Cairo University, Cairo, Egypt; f Department of Pediatric Nephrology, King Faisal Specialist Hospital, Riyadh, Saudi Arabia; g Department of Pediatrics, Umm Alqura University, Makkah, Saudi Arabia; h Department of Pediatric Nephrology, University of Virginia, United States; i Children's Health Center, Department of Pediatrics, International Medical Center, Jeddah, Saudi Arabia

**Keywords:** Lupus nephritis, Atypical hemolytic uremic syndrome, Pericarditis, Plasma exchange, Eculizumab

## Abstract

**Introduction and importance:**

Lupus nephritis is particularly a very concerning occurrence due to the susceptibility for potential renal damage and ultimately renal failure. Cardiac involvement was present as well in the form of pericarditis. Our study reports a case of lupus nephritis that has had a very severe course of fluctuations between relapses and improvements which constantly necessitated an MDT interference at various points.

**Case presentation:**

We report a case of a 13-year-old female patient who presented with a 5-day history of fever, dizziness, joint pain, menorrhagia, convulsions, and visual disturbances. Essential diagnostic tests took place and a diagnosis of lupus nephritis was confirmed.

**Conclusion:**

In conclusion we found that a combination of various treatment modalities and flexibility in decision making in response to changes in the clinical course are vital to treatment success. Utilization of plasma exchange which resulted in an enormous drop in the percentage of fragmented red blood cells (RBCs) from 9.8% to 1.8%.

## Highlights

• The combination of lupus nephritis, atypical hemolytic syndrome, and pericarditis is a very rare and occasional finding.
• A wide range of complications can occur rapidly which require a constant multidisciplinary team availability.
• Utilization of plasma exchange resulted in a significant reduction of the fragmented RBCs percentage from 9.8% to 1.8%.
• Eculizumab usage was essential, the dose used was 900 mg IV weekly for 4 weeks, followed by a fifth dose of 1200 mg IV followed by a 1200 mg IV every 2 weeks after a highly specialized nephrology team involvement.

## 1 Introduction and importance

Among the wide ranged spectrum of pathologic disease manifestations concomitant with systemic lupus erythematosus (SLE), lupus nephritis is particularly a very concerning occurrence due to the susceptibility for potential renal damage and ultimately renal failure [1]. Lupus nephritis initiates a wide range of autoimmune destruction, which may noy be always limited to the kidneys. It is becoming increasingly noticed that lupus nephritis has become a trigger for atypical hemolytic uremic syndrome (aHUS) [2]. Among the other wide range of multisystemic involvement that can be accompanying SLE is that to the heart. Cardiac diseases affect 15–50% of SLE patients [3]. Cardiac involvement typically can include pericarditis, myocarditis, endocarditis and conduction abnormalities [4]. The diagnosis of aHUS is to a great extent a clinically based one which relies to a great extent upon a highly effective multidisciplinary team (MDT) involvement. Our study reports a case of lupus nephritis that has had a very severe course of fluctuations between relapses and improvements which constantly necessitated an MDT interference at various points. This work has been written in accordance with the SCARE criteria [5].

## 2 Case presentation

A 13-year-old Saudi female was transferred from a local hospital as a case of recently-diagnosed systemic lupus erythematosus (SLE) with positive ANA and anti-dsDNA, had presented with an attack of loss of consciousness and convulsions, which was preceded by nausea, vomiting and diarrhea. Prior to admission, she was having palpitations with echocardiography revealing pericarditis. Upon admission, investigations had revealed a severe hemolytic anemia with thrombocytopenia, and nephritis. The patient was in her usual state of good health until 3 months prior to the acute presentation. During these preceding 3 months, she was complaining of worsening loss of appetite, occasional dizziness, and intermittent fever unresponsive to antipyretics, associated with joint pain that was migratory in nature, initially involving the shoulders and knees and later extended to the wrists and ankles. Family history was significant for a second-degree consanguinity in the parents. On admission, the patient had menorrhagia and severe hemolytic anemia with thrombocytopenia for which she needed blood transfusion, as well as hypertension which was managed by amlodipine and labetalol infusion. The patient also had significant renal impairment which was managed by fluid challenge and mega dose(4mg/kg) of furosemide. Moreover, the patient had retinal detachment, albeit reversible.

**Fig. 1:**
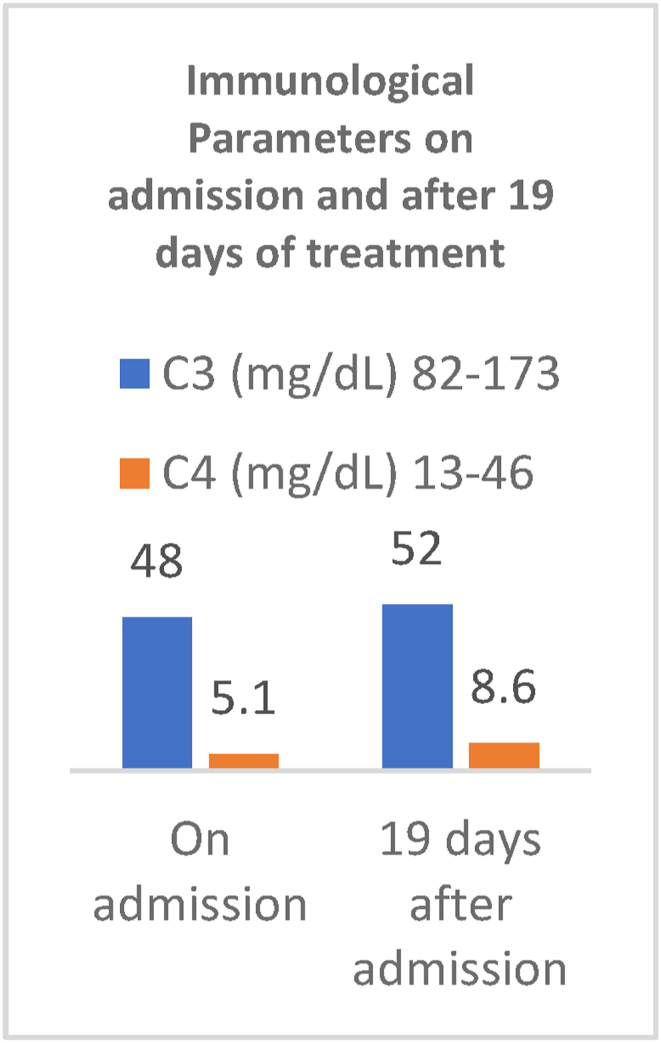
Chart demonstration of the complement levels, on admission and after 19 days of treatment by steroids, plasma exchange, cyclophosphamide, and 1 dose of ecalizumab.

**Fig. 2:**
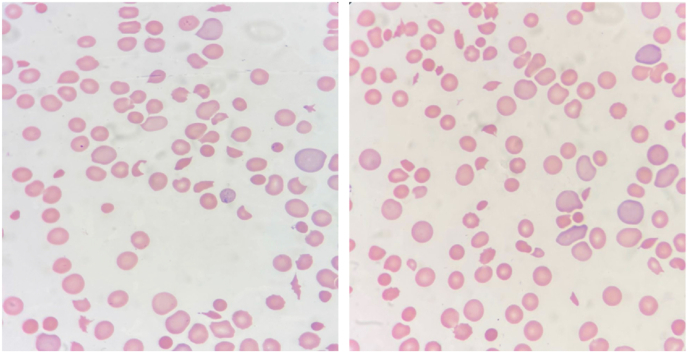
Peripheral blood smear showing shistocytes. Permission from Saudi German

Table 1 summarizes the laboratory findings on admission and during the course of treatment. On admission the patient presented with microangiopathic hemolytic anemia (hemoglobin 8.22 g/dL, hematocrit 24.7%) and thrombocytopenia (platelet 18.6 × 10^9/L), and azotemia (urea 203.51 mg/dL and serum creatinine 2.96 mg/dL), schistocytes of 9.5% were present on the peripheral blood smear (Fig. 2) which was consistent with an ongoing thrombotic microangiopathy (TMA) (schistocytes ≥1%). Interestingly, the patient had also had a severe ADMATS-13 deficiency (0.39 IU/dL). Moreover, the patient had prolonged prothrombin time (21.6 seconds) and an increase in the international normalized ratio (1.63), as well as increase in the D-dimer level (>40 mg/LFEU). However, she had normal levels of activated partial thromboplastin time (36.6 seconds) and fibrinogen (2.52g/L), which indicates that DIC is less likely. She had decreased levels of both serum complement 3 (48 mg/dl) and serum complement 4 (5.1 mg/dl). The patient had decreased albumin (3 g/dL). Moreover, the aspartate aminotransferase levels was high (94 U/L), whereas the alanine aminotransferase was within normal limits (29 U/L). Lactate dehydrogenase was (2755 U/L) and the uric acid level was elevated (14.10 mg/dL). In terms of serum electrolytes, sodium (138.6 mmol/L) and potassium (3.56 mmol/L) levels were normal. Fig. 1 shows level of complements upon admission compared with after multiple regimens of treatment.

**Table 1:** Laboratory findings of the patient on admission, after the 1st dose of eculizumab and 2nd dose of eculizumab.

*Variables*	Normal Values	On admission	3 days after 1st dose	3 days after 2nd dose
***Hematological Parameters***				
*Haemoglobin (g/dL)*	11.5–15.5	8.22	8.38	10
*Platelets (x10 ^ 9/L)*	140–440	18.6	58.9	240.0
*Leukocytes (x10 ^ 9/L)*	5–13	11.6	11	15.10
*Aptt (seconds)*	25–40	36.6	30.3	45.40
*PT (seconds)*	11.7–15.3	21.6	16.5	16.80
*INR*	0.9–1.1	1.63	1.23	1.25

***Renal Parameters***				
*Urea (mg/dL)*	11.0–36.0	203.51	261.51	116.63
*Creatinine(mg/dL)*	0.57–1.11	2.96	4.34	1.71
*Serum Albumin(g/dL)*	3.4–5	3.05	3.3	2.74
*Total proteins serum(g/dL)*	6.4–8.3	7	5.8	5.20

**Fig. 3:**
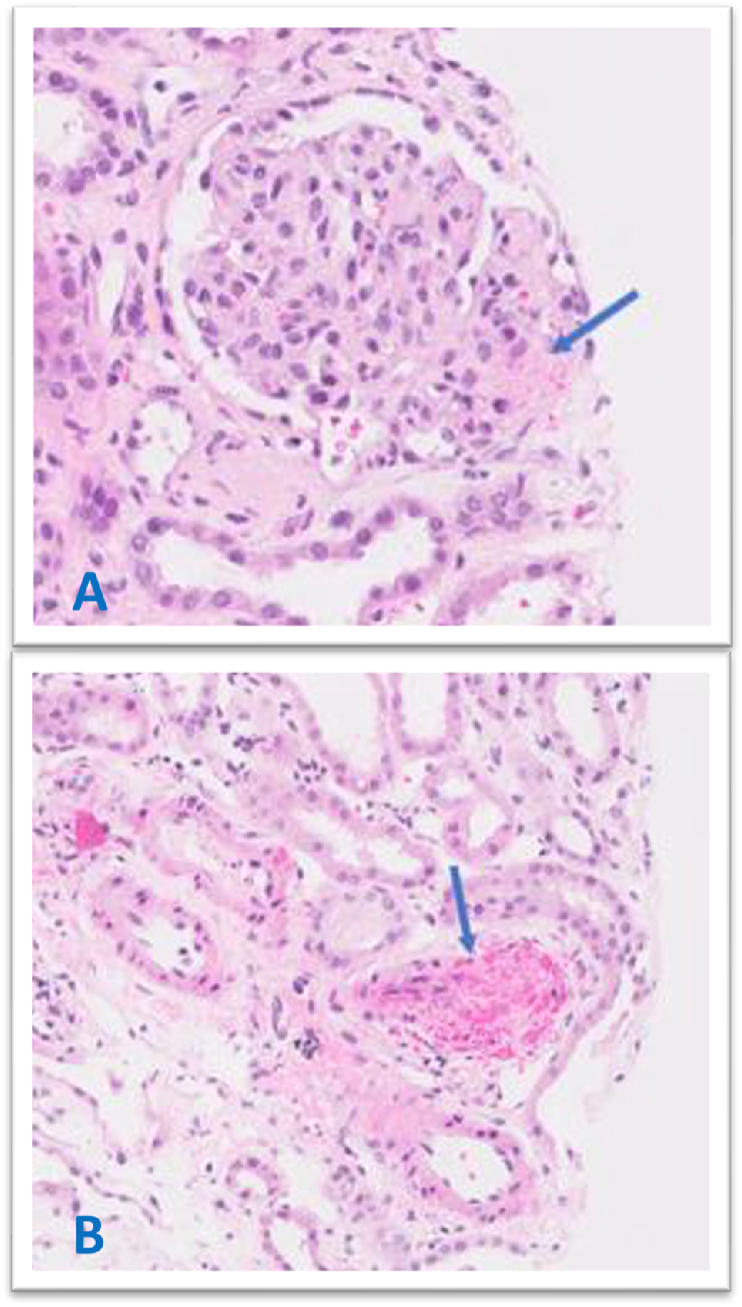
Light microscophy of renal biopsy showing glomuerulus expanded by amorphous materials and fragmented red blood cells **A** (arrow). Small artery occluded by a fibrin thrombus with fibrinoid necrosis of the wall and fragmented red blood cells **B**

**Fig. 4:**
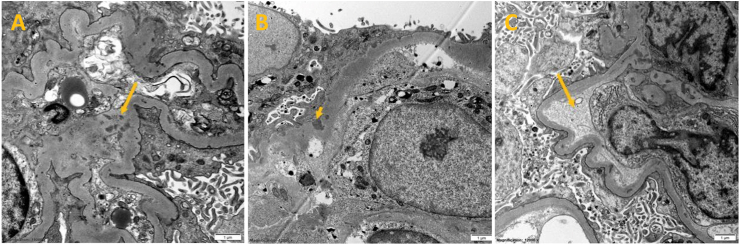
Electron microscophy of renal biopsy histopathology slides. Demonstrating scattered small electron-dense deopsits in the mesangium **(A)** and a few in the glomerular capillary basement membrane **(B)**, as well as segment of glomerular capillary loop with amorphous subendothelial widening and endotheilal cell swelling **(C)**.

Renal biopsy was performed which revealed on light microscopy expanded glomerulus by fragmented red blood cells and amorphous material, as well as occlusion of small artery by fibrin thrombus with fibrinoid necrosis of the wall and fragmented red blood cells as shown in Fig. 3. Electron Microscopy showed deposits in the mesangium and a few in the glomerular capillary basement membrane, as well as amorphous subendothelial widening and endothelial cell swelling of a segment of a glomerular capillary as shown in Fig. 4. On immunofluorescence, IgA, Kappa and Lambda light chain showed prominent tubular cast staining, mild to moderate tubular protein droplet staining was observed for IgG and IgM, diffuse global granular glomerular staining was noted for C1q and C3.

The provisional diagnosis of atypical hemolytic uremic syndrome and SLE was made based on clinical and laboratory findings. During the first five days, the patient was treated with pulse methylprednisolone and IV antibiotics (piperacillin-tazobactam) on the suspicion of an underlying infectious trigger. Two days after admission, multidisciplinary team discussion including nephrologist, rheumatologist, hematologist and pediatric intensivist decided that the patient receives mycophenolate mofetil and the first plasma exchange session which continued for three consecutive days based on experties. On the sixth day, she developed sudden-onset of generalized tonic-clonic seizures and disturbed consciousness which was not controlled inspite of midazolam and prompted endotracheal intubation. She also received her first dose of eculizumab (900 mg). On the seventh day, she received cyclophosphamide treatment. Nevertheless, her renal functions were deteriorating with decrease in urine output for which she received furosemide. Fortunately, three days after receiving the eculizumab the anemia, coagulopathy, thrombocytopenia, as well as the albumin and total serum proteins have shown modest improvement as shown in Table 1, moreover clinically the generalized edema was improving. Five days after the 1st eculizumab dose, plasmapheresis was restarted for five cycles daily in addition to the previous 3 cycles, and she also took another course of pulse steroids due to worsening kidney functions. In spite of that, the patient was still deteriorating being anuric, hypertensive, anemic, and thrombocytopenic. Three weeks after admission, the patient started renal dialysis due to continued oliguria. Five days after starting the renal dialysis, the patient received rituximab, her renal functions, and urine output started to improve. One week after the renal dialysis, the patient's coagulopathy and hypertension started to resolve, and the urine output continued to improve. Five weeks after admission, a skin rash in the form of erythema and erosions appeared on the trunk and then extended to the legs, accompanied by periorbital dusky red erythema. Two days later, the patient developed periorbital maceration and scabbed bullae. However, the rash on the trunk and extremities was improving after discontinuing trimethoprim/sulfamethoxazole. Another dose was of ecalizumab administered five weeks following the 1st dose of eculizumab, based on hematolocal experties to leave a gap following the administration of rituximab. Three days later, the patient showed significant clinical improvement, was interactive with the medical team and with her parents, as well as showed significant improvement in laboratory findings as shown in Table 1. However, after a few days the patient developed high grade fever and sepsis which prevented her from completing the ecalizumab as well as rituximab therapy regimen.

## 3 Clinical discussion

SLE is a multisystemic autoimmune disease with extremely variable clinical presentations, which can range from mild joint and skin involvement to life-threatening renal, hematologic, or neurologic pathology. It is well-established that children and adolescents with SLE are more prone to severe course with earlier disease-damage as compared to adults with SLE [6]. Aside from that, it is not uncommon for patients with SLE to present with thrombotic microangiopathy (TMA), particularly in the form of thrombotic thrombocytopenic purpura (TTP) prior to, during, or after lupus is diagnosed. Indeed, TTP has been shown to result either from a congenital ADAMTS13 enzymatic deficiency or more commonly, autoimmune inhibitors of ADAMTS13 activity [7]. The second subtype of TMA, namely a-HUS has also been associated with SLE. However, it is estimated that the prevalence of a-HUS in the pediatric European population is about seven per million and therefore remains a quite rare disorder, especially in the setting of SLE [8]. Herein a unique presentation of SLE is reported, that possibly lies in the middle of a gray zone between TTP and a-HUS.

We report a case of an adolescent girl with lupus nephritis complicated by TMA with features of both TTP and a-HUS. Not surprisingly, the clinical course was aggressive with multi-organ involvement in the form of cerebral edema, pleural effusion and pericarditis.

Initially, the neurological insult was not well-evident as there was some occasional dizziness and photophobia without any localizing signs. In addition, given the severe nephritis and hypertension on admission (Serum Creatinine 2.96 mg/dL, Total Pr/Cr ratio 5002.65 mg/g, BP 160/100 mmHg), hemolytic anemia (Hb 8.22, Bilirubin 1.12 mg/dL, LDH 2755 U/L) with thrombocytopenia (18.60 × 109/L) and low complement levels (C3 48 mg/dL and C4 5.10 mg/dL), a diagnosis of a-HUS appeared likely. Rauffi et al. describe a case of SLE whose presentation was with complement-mediated HUS as evidenced by persistent hypocomplementemia in the setting of normal ADAMTS-13 level (99%) [9]. In contrast, our case displayed a strikingly low ADAMTS-13 activity (3.9%), which had put the diagnosis of TTP, characterized by ADAMTS-13 level <10%, into consideration [10]. Therefore, treatment had begun with plasmapheresis until Eculizumab was made available. This approach is endorsed by an international consensus by Loirat and colleagues, who advocate initiating therapy by plasma exchange for patients suspected of TTP or a-HUS until a definitive diagnosis can be made [11].

In our patient, there was a good response in renal functions, blood pressure and hematological parameters following the third plasma exchange session. This was similar to the case reported by Samson et al. whose serum Creatinine levels dropped significantly after the third plasma exchange therapy [12]. Notably, however, their patient had a proven diagnosis of TTP and continued to show improvement until complete resolution by the end of the seventh plasmapheresis session [12]. Whereas in our patient, a sudden-onset of generalized tonic-clonic seizures had ensued and continued to recur in spite of seizure-controllers, which ultimately necessitated intubation for an impending respiratory failure. Soon afterwards, treatment with eculizumab had begun, and hematologic parameters were improving. Yet, renal functions continued to deteriorate (BUN and Creatinine) and even after cyclophosphamide was administered.

Some evidence suggests that renal histopathological examination may have a role in distinguishing TTP from a-HUS, as platelet-rich thrombi have been associated with the former while fibrin-rich thrombi with the latter [13,14]. Therefore, following multidisciplinary-team board, a decision for renal biopsy was undertaken; it showed fibrin-rich thrombi occluding most arterioles, some with fragmented RBCs, together with mild mesangial expansion, all of which are indicative of a-HUS. Nevertheless, research is ongoing on this aspect and the literature indicates that definitive distinction between TTP and a-HUS on histopathological grounds alone is not always possible [13,14].

By this stage, multiple immunomodulators were already given, including methylprednisolone and hydroxychloroquine. Regardless, acute kidney injury had ensued and the patient was put on dialysis for a total of eleven days. In order to allow for spacing of the biologic agents, Rituximab was given three days later, corresponding to three-weeks interval from the first dose of Eculizumab. Meanwhile, plasmapheresis was resumed and yielded a waxing-and-weaning clinical and laboratory response. The patient's total length of stay in the pediatric intensive care unit (PICU) was 74 days, and her course was complicated by sepsis. Owing to the clinical deterioration of the patient with development of septic shock, the scheduled regimen of eculizumab and rituximab could not be followed.

Our case represents a complex association of two well-recognized thrombotic microangiopathy syndromes (TTP and HUS), that were likely triggered by a combination of genetic and acquired predisposition in the form of SLE.

In one end of the TMAs spectrum, atypical HUS arises from a congenital or acquired overactivation of the alternative complement pathway [15]. After the advent of a novel targeted treatment-modality in the last decade (eculizumab), the prognosis for a-HUS patients has witnessed a dramatic improvement. However, a growing body of evidence indicates that the response to eculizumab is dictated by the underlying complement defect. Indeed, certain forms of hereditary HUS have shown inadequate to poor response to eculizumab, including complement-factor H (CFH), membrane cofactor protein (MCP), complement-factor I (CFI) and diacylglycerol kinase ε (DGK- ε) [15,16]. Currently, more genetic variants are being investigated, adding to the growing knowledge on a-HUS pathophysiology. Similarly, our patient may had been harboring a unique genetic variant and/or antibodies.

On the other end of the spectrum, TTP may not be as benign as it is thought. While acute kidney injury is an extremely rare phenomenon with TTP, SLE-driven TMAs may compromise patients’ renal functions and worsen their prognosis. Indeed, studies indicate that TTP in association with SLE has a high mortality that ranges from 34 to 62.5% [17].

In conclusion, it is imperative for clinicians caring for SLE patients to recognize the propensity of these patients to developing microangiopathic hemolytic anemias that without prompt diagnosis and treatment, can be rapidly-progressive with fatal sequelae.

## 4 Conclusion

In conclusion we found that a combination of various treatment modalities and flexibility in decision making in response to changes in the clinical course are vital to treatment success. In our patient various treatment modalities were used and among the most effective ones was the utilization of plasma exchange which resulted in an enormous drop in the percentage of fragmented red blood cells (RBCs) from 9.8% to 1.8%. Additionally, the usage of Eculizumab was optimized after a highly specialized nephrology team involvement in a mutually agreed on protocol aiming to optimize the clinical output and reduce mortality and morbidity risks in our patient.

## Patient consent

Written informed consent was obtained from the patient for publication of this case report and accompanying images. A copy of the written consent is available for review by the Editor-in-Chief of this journal on request.

## Provenance and peer review

Not commissioned, externally peer reviewed.

## Statement of ethics

This study complies with internationally accepted standards for research practice reporting.

## Ethical approval

Ethical approval has been given by the Institutional Review Board (IRB) of our institution, Saudi German Hospital, Jeddah, Saudi Arabia.

## Sources of funding

No funding for this research.

## Author contribution

Drafting of the manuscript: Ahmed Hafez Mousa, Reham Salama Alshaltoni, Amani Mohsen Kiswany, Critical revision of the manuscript for important intellectual content: Ahmed Hafez Mousa, Reham Salama Alshaltoni, Amani Mohsen Kiswany, Abdullah Alghobaishi, Abdullah Baothman, Abeer Amin, Hanan Adem, Yasir Eisa, Burhan Edrees.

## Registration of research studies

Name of the registry:
Unique Identifying number or registration ID:
Hyperlink to your specific registration (must be publicly accessible and will be checked):

## Consent

This is a single case report with no identifiable patient information are included in this case report. An Institutional Review Board (IRB) approval however was obtained from Saudi German Hospital, Jeddah, Saudi Arabia prior to the conduction of the report.

Written informed consent was obtained from the patients parents for publication of this case report and accompanying images. A copy of the written consent is available for review by the Editor-in-Chief of this journal on request.

## Guarantor

Ahmed Hafez Mousa, corresponding author of the manuscript, accept full responsibility for the work and the conduct of the study, had access to the data, and controlled the decision to publish.

## Declaration of competing interest

No conflicts of interest.
